# The Role of Glucosamine-Induced ER Stress in Diabetic Atherogenesis

**DOI:** 10.1155/2012/187018

**Published:** 2012-02-23

**Authors:** Daniel R. Beriault, Geoff H. Werstuck

**Affiliations:** ^1^Thrombosis and Atherosclerosis Research Institute, McMaster University, 237 Barton Street East, Hamilton, ON, Canada L8L 2X2; ^2^Department of Biochemistry and Biomedical Sciences, McMaster University, Hamilton, ON, Canada L8N 3Z5; ^3^Department of Medicine, McMaster University, Hamilton, ON, Canada L8N 3Z5

## Abstract

Cardiovascular disease (CVD) is the major cause of mortality in individuals with diabetes mellitus. However the molecular and cellular mechanisms that predispose individuals with diabetes to the development and progression of atherosclerosis, the underlying cause of most CVD, are not understood. This paper summarizes the current state of our knowledge of pathways and mechanisms that may link diabetes and hyperglycemia to atherogenesis. We highlight recent work from our lab, and others', that supports a role for ER stress in these processes. The continued investigation of existing pathways, linking hyperglycemia and diabetes mellitus to atherosclerosis, and the identification of novel mechanisms and targets will be important to the development of new and effective antiatherosclerotic therapies tailored to individuals with diabetes.

## 1. Introduction

Worldwide, cardiovascular disease (CVD) is the leading cause of premature death in both men and women. Risk factors for CVD include abnormal lipid levels, smoking, hypertension, abdominal obesity, stress, sedentary lifestyle, and diabetes mellitus [1]. While the incidence of CVD has declined in many developed countries, this trend is expected to reverse in the near future [2]. This is largely due to the dramatic, worldwide increase in the incidence of diabetes mellitus. Driven by changes in lifestyle and an escalating rate of obesity, the number of individuals with diabetes may already be as high as 350 million [3, 4]. Diabetes mellitus is a major, independent risk factor for cardiovascular disease (CVD), and individuals with diabetes are 2 to 3 times more likely to die from CV causes than people with no history of diabetes, even after controlling for other CV risk factors [5–9]. These individuals are also at increased risk of diseases that are associated with CVD and atherosclerosis including hypertension and renal failure. Ultimately, this translates to a CV mortality rate in diabetic patients of approximately 75% [6, 7]. The increasing incidence of diabetes means that the global burden of this chronic disease on health care resources will continue to rise for the foreseeable future. 

It is not clear why individuals with diabetes are predisposed to CVD. Recent reports from clinical trials examining the effects of intensive blood glucose lowering on CV risk, including ACCORD (Action to Control Cardiovascular Risk in Diabetes) [10], ADVANCE (Action in Diabetes and Vascular Disease: Preterax and Diamicron MR Controlled Evaluation) [11], UKPDS (United Kingdom Prospective Diabetes Study) [12], and VADT (Veterans Affairs Diabetes Trial) [13], suggest that the relationship between hyperglycemia and CVD is complex. Despite a vast amount of research, currently available treatments show only limited CV benefit and CVD continues to be the major cause of mortality.

There is a strong correlation between hyperglycemia and both micro- and macrovascular disease [14–18]. The negative effects of elevated glucose levels on vascular function can include decreased proliferation of endothelial cells, the impairment of some parameters of vascular responsiveness, and increased endothelial programmed cell death [19–21]. It is well established that aggressive blood glucose lowering significantly decreases the incidence and severity of microvascular disease including retinopathy, renal failure and peripheral nerve dysfunction [14, 15]. Recent evidence suggests that increased glycemic control also correlates with a reduction in macrovascular disease; however, the relationship between glucose lowering and a decrease in CVD has been much more difficult to demonstrate [15]. Several explanations have been put forth to rationalize the inability of clinical trials to demonstrate a strong improvement in cardiovascular outcomes through glycemic control including the possibilities that the trials were underpowered, were too short in duration, or were too focused upon fasting glucose rather than postprandial glucose levels. Alternatively, these findings may indicate that the quality of glycemic control presently achievable is insufficient to be effective in protecting against macrovascular disease. Therefore, even short-term deviations in the control of blood glucose may promote vascular dysfunction.

The pathophysiology of T2D-associated CVD is further complicated by multiple risk factors, collectively known as the metabolic syndrome, that commonly accompany chronic hyperglycemia. The metabolic syndrome is clinically defined as a combination of abdominal obesity, insulin resistance (prediabetes), atherogenic dyslipidemia, and hypertension [22]. The metabolic syndrome is a major cause of morbidity and mortality with cardiovascular disease being the primary clinical outcome [22]. Other complications can include respiratory difficulties, chronic skeletal muscle problems, gall bladder disease, infertility, hepatic steatosis, circulatory problems, and certain cancers [23, 24].

Therefore, while a role for hyperglycemia in the development and progression of atherosclerosis is supported by a great deal of basic research, the clinical role of elevated glucose levels in macrovascular disease is less clear. Furthermore, despite a great deal of research, the mechanisms that may link high glucose concentrations to the molecular and cellular pathways of disease development are not fully understood. This paper will focus on potential direct proatherogenic consequences of hyperglycemia.

## 2. Mechanisms and Pathways Linking Diabetes and Hyperglycemia to CVD and Accelerated Atherosclerosis

Several mechanisms have been proposed to explain the proatherogenic effects of diabetes and hyperglycemia. In general these have focused upon the intracellular effects of elevated levels of glucose, and the increased availability of glucose metabolites, in cells of the vascular wall. There is evidence that hyperglycemia is associated with increased aldose reductase activity that can lead to increased consumption of NADPH and depletion of GSH levels resulting in elevated levels of reactive oxygen species (ROS) and subsequent cellular damage [25, 26]. Glucose-induced PKC activation has been implicated in decreased endothelial vasodilation [27] and increased production of ROS [28] that could contribute to endothelial dysfunction. It has also been proposed that the conversion of sorbitol to 3-deoxyglucosone can feed into the production of advanced glycation endproducts (AGEs). AGES are formed through a nonenzymatic process, known as the Maillard reaction, involving the reaction of the aldehyde groups of reducing sugars with the amino groups of proteins [29, 30]. There are several potential pathways where AGE-modified proteins could be damaging; the formation of AGEs may alter protein function [31], disrupt the extracellular matrix [31, 32], and/or lead to the modification of lipoprotein particles thereby increasing their atherogenicity. However the predominant vascular effect of AGEs appears to occur through their interaction with RAGE (receptor for AGE) found on macrophages and endothelial and smooth muscle cells [33–35]. The AGE-RAGE interaction triggers a signal transduction cascade that results in the production of intracellular ROS that can initiate an inflammatory response [36, 37]. 

While preclinical evidence supports a causative role for oxidative stress in atherogenesis [38–41], virtually every well-controlled clinical trial has failed to show a cardiovascular benefit in diabetic patients receiving antioxidant supplements [42–46]. There are several ways to rationalize this apparent paradox by questioning: the specific antioxidants tested, the doses prescribed, and/or the power and duration of the trials themselves. However, these clinical observations may be indicative of the existence of other important molecular mechanisms or pathways that play a causative role in diabetic atherogenesis in addition to oxidative stress.

### 2.1. The Hexosamine Pathway

 Conditions of hyperglycemia also result in the shunting of excess intracellular glucose through the hexosamine biosynthetic pathway (HBP). In a typical cell, under normoglycemic conditions, 1 to 3% of total glucose will be converted to glucosamine-6 phosphate by the enzyme glutamine:fructose-6 phosphate amidotransferase (GFAT) [47]. When intracellular glucose levels rise, flux through this pathway increases. Furthermore, increased GFAT expression and activity have been reported in tissues from humans with diabetes [48]. The net result is an elevated intracellular concentration of glucosamine. This effect has been observed in cultured cells challenged with elevated concentrations of glucose as well as in vascular and hepatic tissues of hyperglycemic animals [49–52].

Increased hexosamine pathway flux has been implicated in several diabetes-associated complications including insulin resistance [47, 53] and pancreatic *β* cell death [54], as well as atherosclerosis [55]. The molecule mechanisms that underlie the pathogenic effects of increased HBP flux are not fully understood. Most research has focused upon the role of UDP-N-acetylglucosamine (UDP-GlcNAc), the end-product of the HBP pathway and a substrate for both O- and N-linked protein glycation, as a causative agent. It is well established that elevated glucosamine concentrations drive the O-linked glycosylation of proteins including transcription factors [56] and nuclear pore proteins [57], as well as signaling factors [58] which potentially alters their function, stability, and/or activity. Specifically, studies have suggested that O-glycosylation may regulate transcription, plasma lipids, and gluconeogenesis by modulating the activation of RNA polymerase II [59], angiopoietin-like protein 3 [60], FoxO1, and CRTC2 [61, 62], respectively (further reviewed in [63]). Glucosamine has been shown to desensitize insulin-stimulated glucose uptake in both adipocytes [47] and skeletal muscle cells [64], probably by inhibiting the translocation of the glucose transporter, GLUT4, to the cell surface [65]. In addition, increased hexosamine pathway activity can promote the transcription of proinflammatory and prothrombotic factors including TGF-*α*, TGF-*β*, and PAI-1 [66–68]. Therefore, the hyperglycemia-induced O-GlcNAc modification of proteins may change gene expression patterns and alter the function of specific factors that contribute to a proatherogenic, prothrombophilic vascular environment. More studies are required to test this theory and to precisely determine the factors and downstream pathways that may be involved in the acceleration of vascular disease.

 UDP-GlcNAc is also a substrate for N-linked protein glycation that occurs in the endoplasmic reticulum (ER). N-glycosylation is an important posttranslational modification of nascent proteins that is critical for proper protein folding [69]. Mutations in asparagine residues of specific proteins, which are critical for N-glycosylation, result in disrupted folding, secretion and/or activity [70–72]. Tunicamycin, a UDP-GlcNAc antagonist, has been shown to inhibit N-glycosylation and activate the cell's quality control mechanism: the unfolded protein response [73–75]. Ultimately, disruptions in the N-glycation process can lead to an accumulation of unfolded/misfolded proteins in the ER that perturb the ER homeostatic balance; this is known as “ER stress.” An additional intracellular effect of glucosamine, which has not been investigated in the context of diabetes and atherosclerosis, is its ability to promote the accumulation of unfolded proteins in the ER, thereby leading to ER dysfunction and cell injury [75–78].

## 3. The Endoplasmic Reticulum and the Unfolded Protein Response

In a typical eukaryotic cell, the ER is responsible for the proper modifying, folding, and trafficking of approximately one-third of all proteins. ER-localized modifications of nascent proteins include disulfide bond formation and N-linked glycosylation, which are critical to protein folding [69]. Unfolded/misfolded proteins are directed to undergo ER-associated degradation (ERAD), and, under physiological conditions, the ER is able to maintain a homeostatic balance between folded and misfolded proteins [79]. When the ER processing capacity is overwhelmed, unfolded/misfolded proteins accumulate and disrupt the ER homeostatic balance; this is known as ER stress.

**Figure 1:**
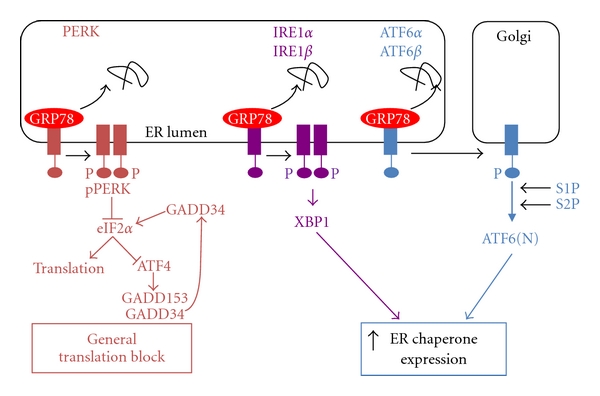
The unfolded protein response to endoplasmic reticulum stress. ER stress occurs when the capacity of the ER to process/fold proteins is exceeded by the load of nascent proteins entering the ER. The function of the UPR is to reestablish ER homeostasis by decreasing protein flux into the ER (translation block) while increasing the folding capacity of the ER (increased chaperone expression). Conditions of ER stress lead to the dissociation of ER chaperone GRP78 from the trans-ER-membrane signaling factors PERK, IRE1, and ATF6, resulting in their activation. Activated PERK phosphorylates and inhibits the activity of eIF2*α*, an essential factor in general protein translation. PERK is also involved with the downstream activation of transcription factors including ATF4 and GADD153. Activated IRE1 assists in the alternative splicing of XBP-1 resulting in the translation of a transcription factor, XBP-1, which is involved in upregulation of the expression of ER chaperones. Activated ATF6 translocates to the Golgi where proteases S1P and S2P release an N-terminal transcription activation domain that works in concert with XBP-1 to upregulate ER chaperone expression.

Traditional ER-stress-inducing agents are known to disrupt protein folding by interfering with disulphide bond formation (dithiothreitol) [80], ER Ca^2+^ balance (A23187, thapsigargin) [81], ER membrane structure (palmitate, unesterified cholesterol) [82, 83] or by blocking protein N-glycosylation (tunicamycin) [84]. Conditions of ER stress activate the unfolded protein response (UPR) which functions to restore ER homeostasis (Figure 1). The UPR is a three pronged signaling cascade that is initiated by trans-membrane ER proteins known as inositol-requiring enzyme (IRE)-1, activating transcription factor (ATF)-6, and PKR-like ER kinase (PERK) [85]. Initiation of these pathways alleviates ER stress by decreasing protein synthesis, increasing ER chaperone levels, and facilitating degradation of irreversibly misfolded proteins. Under conditions of chronic ER stress, upregulation of pathways involved in lipid accumulation (SREBP) and inflammation (NF-*κ*B) can occur [49, 86–88]. If the UPR is unable to restore ER homeostasis, proapoptotic signaling factors (i.e., GADD153/CHOP) are upregulated to initiate programmed cell death [89].

### 3.1. ER Stress and Atherogenesis

There is increasing experimental evidence in support of a direct and causative role for ER stress in the development and/or progression of atherosclerosis. First, several independent risk factors for CVD, including hyperglycemia [49], hyperhomocysteinemia [7, 88], obesity [90], and elevated levels of palmitate [91] and unesterified cholesterol [92], have been associated with ER stress, suggesting that this pathway may represent a common or unifying mechanism of accelerated atherogenesis [93, 94]. Secondly, activation of the UPR has been noted at all stages of atherosclerotic development, from a fatty streak to an advanced occlusive plaque [95]. Third, conditions of ER stress can activate/dysregulate metabolic pathways that are directly involved in the development of atherosclerotic lesions. ER-stress-inducing agents promote lipid accumulation by activating the sterol regulatory element binding proteins (SREBPs), which are transcription factors that control lipid biosynthesis and uptake [88, 96, 97]. ER-stress-inducing agents also activate NF-*κ*B, the transcription factor responsible for promoting inflammatory gene expression [98, 99]. Finally, ER stress has been shown to activate caspases and promote apoptosis of human aortic endothelial cells and other cell types [100, 101]. Together, lipid accumulation, inflammation, and endothelial apoptosis are the hallmark features of atherosclerosis [102, 103].

### 3.2. Glucosamine-Induced ER Stress

Our lab has recently overexpressed the HBP rate limiting enzyme, GFAT, using an adenoviral expression system in cell culture and measured a significant increase in UPR gene expression and downstream effects of ER stress including lipid accumulation, inflammatory gene expression, and apoptotic signaling under hyperglycemic conditions [86]. We have shown that addition of exogenous glucosamine, or increased endogenous production of glucosamine, can disrupt the capacity of the ER to process nascent proteins and initiate an ER stress response. Furthermore, this effect has been observed in cell types that are relevant to the development of atherosclerosis, including human aortic smooth muscle cells, monocyte-derived macrophages, and HepG2 cells [49, 50, 100, 101]. Thus, elevated levels of glucosamine may play an important role in ER and cellular dysfunction associated with atherogenesis.

It is not known how increased concentrations of glucosamine (but not mannose) disrupt protein folding in the ER. UDP-N-acetylglucosamine is an essential substrate for both O- and N-linked protein glycosylation, and protein glycosylation is an important step in the proper folding of many proteins [69]. It is known that elevated concentrations of glucosamine increase levels of O-linked protein glycosylation [49] and alter N-linked glycosylation patterns of specific proteins including ApoB100 [104]. It is possible that either of these effects could promote ER stress. In cultured HepG2 cells, our lab has shown that PUGNAc, an inhibitor of O-GlcNAcase, increases protein-O-GlcNAc levels but does not promote ER stress [49]. This may suggest that glucosamine-induced ER stress is caused by free and not protein O-linked glucosamine. We hypothesize that increased levels of glucosamine, or a derivative of glucosamine, may interfere with a step in the N-linked glycation of proteins resulting in the production of misfolded proteins and the activation of the UPR.

 Elevated levels of glucosamine and glucosamine-induced ER stress have been previously implicated in acquired insulin resistance [47, 53, 105, 106]; however, there is some controversy to whether this effect is physiologically relevant in humans. Incubation of relatively high concentrations of glucosamine (1–10 mmol/L) in adipose, muscle, or endothelial cell cultures has been implicated in impaired insulin action [106–109]. Furthermore, high levels of intravenously injected glucosamine (plasma concentrations of 0.5–1.8 mmol/L) in both animals and humans have also been shown to cause insulin resistance [110, 111]. The recommended daily dose of oral glucosamine supplements, commonly taken to treat joint pain, are far lower (plasma concentrations of ~3 *μ*mol/L), and data suggest that these supplements have no effect on insulin sensitivity [112, 113]. Additional studies will be required to determine the effects of chronic hyperglycemia on endogenous, intracellular levels of glucosamine and possible effects on insulin resistance.

## 4. Hyperglycemia, ER Stress, and Accelerated Atherosclerosis

**Figure 2:**
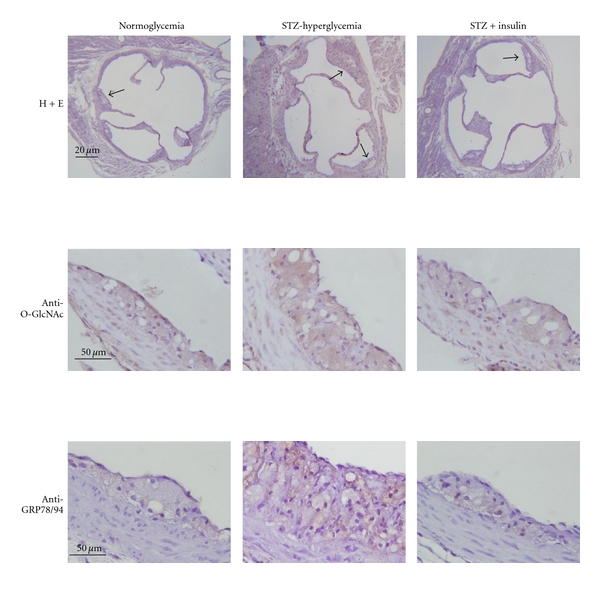
Analysis of aortic root from normoglycemic, STZ-injected hyperglycemic, and STZ-injected insulin-treated ApoE^−/−^ mice. Hyperglycemic mice show increased vascular O-linked GlcNAc, elevated levels of ER stress markers (GRP78/94), and significantly accelerated atherosclerotic lesion development, relative to normoglycemic controls. Normalization of glucose levels with insulin attenuates O-GlcNAc accumulation, ER stress, and atherogenesis.

 To investigate the molecular mechanisms that link hyperglycemia to atherosclerosis, we have established a model in which we inject ApoE^−/−^ mice with multiple low doses of streptozotocin (STZ) [49, 50, 114]. Using this model we have observed a correlation between hyperglycemia, the accumulation of glucosamine in the artery wall, vascular ER stress, and accelerated atherogenesis [49] (Figure 2). Significantly, ER stress levels in the endothelium of hyperglycemic mice increase prior to the development of the atherosclerotic lesions, a result that is consistent with ER stress playing a causative role in lesion development [50]. In addition, accelerated lesion development is observed in these diabetic mice before the onset of dyslipidemia, suggesting that hyperglycemia is sufficient to independently promote the activation of proatherogenic processes [49].

In a direct test of the atherogenic potential of glucosamine, we have recently found that ApoE^−/−^ mice given drinking water containing 5% glucosamine (w/v) for 7 weeks have significantly increased vascular and hepatic ER stress levels in addition to larger atherosclerotic lesions than mice given regular water or water containing 5% mannitol (w/v) [115]. This is consistent with a report from Tannock et al. who found that 5 weeks of glucosamine supplementation increased lesion size in LDL-receptor-deficient mice [116]. Our data suggests that glucosamine-induced ER stress plays a direct and causative role in accelerated atherogenesis. 

## 5. ER Stress in Patients with Metabolic Syndrome

 There is ample evidence *in vitro* and in animal models to support a role for ER stress in the development and complications of diabetes. Recently, small clinically relevant studies involving humans with metabolic syndrome have been carried out. Patients with diabetic nephropathy have been shown to have increased GFAT expression in glomerular epithelial and mesangial cells and that GFAT is expressed in most tissues involved in diabetic complications [48, 117]. Pancreatic beta cells isolated from type 2 diabetics have been shown to have marked expression of ER stress markers [118] and increased susceptibility to ER stress compared to nondiabetic controls [119] and that ER stress may contribute to IL-1*β* production, mild islet inflammation [120], and *β*-cell failure [118]. Our lab has recently shown that isolated leukocytes from human subjects with metabolic syndrome, compared to healthy subjects, have elevated levels of ER stress markers and that there is an association between acute and chronic dysglycemia and ER stress in humans [86]. Each of these trials is consistent with diabetes-associated ER stress playing a clinically relevant role in the pathogenesis of diabetic complications.

## 6. Targets for Potential Therapeutic Intervention

**Figure 3:**
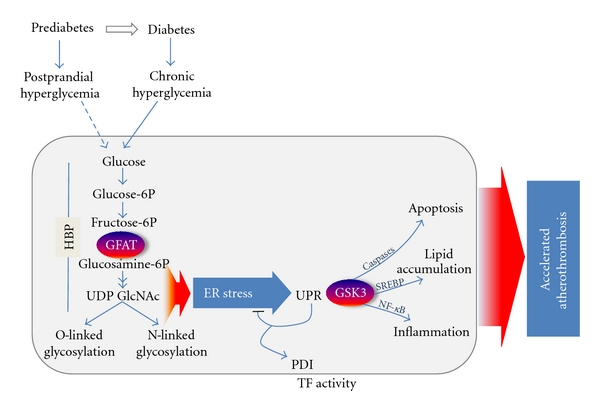
Working model of diabetes-associated accelerated atherothrombosis. Chronic hyperglycemia leads to increased flux through the hexosamine biosynthesis pathway (HBP) resulting in accumulation of UDP-N-acetylglucosamine (UDP-GlcNAc), a substrate for both O- and N-linked protein glycosylation, as well as increased levels of ER stress. Disruptions in ER homeostasis lead to activation of the unfolded protein response (UPR) and downstream effects including activation of glycogen synthase kinase (GSK)-3. Our results suggest that ER-stress-induced GSK-3 induces proatherogenic processes leading to the accelerated development of atherothrombosis.

The identification of a role for ER stress and/or the UPR in the development and progression of diabetes-associated atherosclerosis is significant, not only because it gives us insight on an important disease process, but also because it illuminates novel potential targets for therapeutic intervention (Figure 3). Efforts to develop strategies to manipulate the UPR have already begun, especially with respect to other diseases and disorders in which ER stress is thought to play a role. At least three general approaches have been used to address this problem. The first involves reducing the levels of ER stress directly by relieving the load of misfolded proteins though the addition of chemical chaperones such as 4-phenylbutyric acid (4-PBA), taurine-conjugated ursodeoxycholic acid (TUDCA), or dimethyl sulfoxide (DMSO) [121–123]. The mechanisms by which these small molecules function to reduce ER stress levels are not well defined. However, such strategies have been shown to be effective *in vitro* and *in vivo,* and 4-phenylbutyric acid has been shown to attenuate atherosclerosis in an ApoE^−/−^ mouse model [124]. A second strategy is to augment the protective aspects of the endogenous UPR. This has previously been accomplished through the over-expression of ER-resident protein chaperones including GRP78. The third approach is to target some of the detrimental downstream effects of ER stress. Examples of this strategy include the use of salubrinal which inhibits the phosphatase GADD34 from reactivating eIF2*α*, thereby maintaining the PERK pathway-induced translation block (Figure 1). Other possible targets for intervention would include proinflammatory and/or proapoptotic factors such as ASK1, p38MAPK, or GADD153/CHOP. Indeed, GADD153/CHOP-deficient mice are resistant to accelerated atherosclerosis [125, 126]. Recently we have identified glutamine:fructose-6-phosphate amidotransferase (GFAT) and glycogen synthase kinase (GSK)-3 as two enzymes involved in ER stress and potential targets for therapeutic intervention. 

### 6.1. Glutamine:Fructose-6-phosphate Amidotransferase (GFAT)

The potential role of glucosamine-induced ER stress in diabetic atherogenesis highlights the importance of glutamine:fructose-6-phosphate amidotransferase (GFAT), the rate-limiting enzyme in the conversion of glucose to glucosamine, also known as the hexosamine pathway [127, 128]. A central role for GFAT activity in the ER stress pathway is supported by our finding that inhibition of GFAT attenuates glucose-induced ER stress [49] and that overexpression of GFAT is sufficient to promote ER stress in HepG2 cells cultured in normoglycemic conditions [86]. We are currently developing strategies to modulate GFAT activity *in vitro* and in our mouse models. These tools will be used to investigate the potential effects of regulating GFAT activity on the UPR and on activation of proatherogenic processes.

### 6.2. Glycogen Synthase Kinase (GSK)-3

The mechanisms that link conditions of ER stress to the activation of proatherogenic pathways are not known. GSK-3*α* and *β* are two homologous serine/threonine kinases that are involved in a diverse number of intracellular signaling pathways [129]. We have shown using small molecule inhibitors and GSK-3*α* ^−/−^ and GSK-3*β* ^−/−^ mouse embryonic fibroblasts that GSK-3-deficiency attenuates ER-stress-induced apoptosis and lipid accumulation [114, 130–132]*. In vivo* we have shown that hyperglycemic mice fed a diet supplemented with valproate, a compound that inhibits GSK-3 activity, have reduced hepatic GSK-3 activity and reduced lesion volume at the aortic sinus [114]. Together, the above findings support our hypothesis that glucosamine-induced ER stress plays a role in accelerated atherogenesis and identifies GSK-3 as a potential target for antiatherogenic therapy. The limitation of targeting GSK-3 arises from the central role that this kinase plays in many diverse metabolic processes and the possibility of detrimental side-effects of small molecular inhibitors [118].

## 7. Conclusions

Because of the cardiovascular risks of diabetes and the increasing prevalence of type 2 diabetes, it is essential that we further our knowledge of how and why diabetes mellitus and hyperglycemia promote cardiovascular disease. Currently, and for the near future, the primary strategy for managing cardiovascular disease in the diabetic population will be through the control of hyperglycemia and through the treatment of associated complications such as hypertension and dyslipidemia using established medications such as ACE inhibitors, statins, and fibrates.

The continued identification and investigation of pathways linking hyperglycemia and diabetes mellitus to atherosclerosis is important to the development of new and effective antiatherosclerotic therapies that are tailored to individuals with diabetes. A great deal of research has been focused upon the role of hyperglycemia in the development and progression of atherosclerosis in cell culture and animal model systems. Several mechanisms have been identified that appear to link glucose to proatherogenic processes. The most promising of these, the polyol pathway, PKC activation, the hexosamine pathway, and the AGE-RAGE interaction, show potential and are actively being evaluated as targets for putative antiatherogenic therapies. Thus far, however, all interventions targeting the effects of hyperglycemia, including direct glucose lowering, appear to show greater effect in the treatment of microvascular complications than in the control of macrovascular disease. This is likely due, at least in part, to the complexities of atherosclerosis and current limitations of the animal models available to researchers who study the development and progression of atherosclerosis. Additional studies are obviously required to further our understanding of this important disease.
